# Financial indebtedness and suicide: A 1-year follow-up study of a population registered at the Swedish Enforcement Authority

**DOI:** 10.1177/00207640211036166

**Published:** 2021-08-02

**Authors:** Yerko Rojas

**Affiliations:** School of Social Sciences, Södertörn University, Huddinge, Sweden

**Keywords:** Suicidal behavior, debt repayment, attachment of earnings, property foreclosure, eviction, Sweden

## Abstract

**Background:**

Economic hardship is an established suicidogenic factor. However, very little is known about whether financial difficulties in terms of debt problems, specifically, is related to suicide. This would seem to be an important research gap, not least at a time when the repercussions of the global financial crisis are still being felt by many people.

**Aims:**

This study sets out to examine whether experiencing financial indebtedness is related to suicide.

**Methods:**

For this purpose, people aged between 18 and 64 with a registration date for a debt in the Swedish Enforcement Authority register between 2015 and 2017 (*n* = 180,842) are followed up for a 1-year period for death by suicide and compared with a sample from the general Swedish population (*n* = 928,265). The analysis is based on penalized maximum likelihood logistic regressions.

**Results:**

Those who had experienced financial indebtedness were two and a half times more likely to commit suicide than those who had not lived through this experience (OR = 2.50), controlling for several demographic, socio-economic, and mental health conditions prior to the date of the registration at the Enforcement Authority.

**Conclusion:**

Debt repayment problems have a significant and detrimental impact on individuals’ risk of committing suicide, even when several other socioeconomic risk factors are controlled for. The results reinforce the importance of ongoing attempts to remove the issue of debt problem from its status as a rather hidden suicidogenic risk factor.

## Introduction

The evidence base for linking debt with suicide is not particularly strong (Haw et al., 2015). The amount and variety of studies on the subject is limited (Richardson et al., 2013; Turunen & Hiilamo, 2014). For example, capturing and following representative and sizeable groups of individuals who have experienced financial hardship using traditional surveys has proven difficult (Oksanen et al., 2016; Webley & Nyhus, 2001) – and there is a lack of clarity as to how financial indebtedness should be understood, that is, when debt becomes a problem (European Commission, 2008; Haw et al., 2015). This is quite troublesome, not least at a time when the COVID-19 pandemic is unleashing the largest contraction in economic activity since the Great Depression (United Nations Development Programme, 2020), the repercussions of the latest global financial crisis are still being felt by many people (Di Leo et al., 2018), and voices are being raised to make ‘tackling debt and financial distress’ a new strand in suicide prevention (Money and Mental Health Policy Institute, 2016). After all, having a well-defined problem and a vulnerable group to target is a prerequisite of any selective prevention strategy (WHO, 2014).

To further pinpoint the relation between debt and suicide, this study sets out to examine the extent to which an enhanced suicidogenic risk can be located to the registration date for a debt at the Swedish Enforcement Authority. There are at least three important advantages for studying the relationship in this way: (a) theoretically, the problems in the debt repayment phase of the indebtedness process are those suggested to be suicidogenic (Inoue et al., 2012; Lester & Yang, 2012); (b) examining all cases in which a non-payment of debt that has been officially registered falls well within the administrative model of studying indebtedness (Turunen & Hiilamo, 2014) and is also what official governmental reports have described as the most reliable measure of over-indebtedness available in Sweden (SOU, 2013:78). (c); other countries, such as the Nordic countries, Great Britain, and The Netherlands, have been found to have a similar legal system for countering financial indebtedness (SOU, 2013:78), suggesting that the results from following this specific group of debtors may be of value beyond the boundaries of Sweden.

This study utilizes the opportunity provided by the Microdata Online Access system at Statistics Sweden to link and analyze data from different nationwide registers, including data on debtors’ socioeconomic, sociodemographic, criminal, and mental health status prior to their debt problems, allowing us to adjust for confounding factors and generalize the findings in an unprecedented manner. All women and men aged 18 to 64 years with a registration date for a debt at any point between 2015 and 2017 in the Enforcement Authority register have been followed up for death by suicide and compared to a sample from the general Swedish population.

### Non-payment of debt and suicide

Throughout history, debt has been enforced using varying degrees of punishment including, and most notoriously, imprisonment (Di Leo et al., 2018). The principle that we must all repay our debts can be traced as least as far back as ancient Greece, with the image of a dying Socrates (of Plato’s Phaedo) who uses his last words to implore Crito to repay his debt to Asclepius (Di Leo et al., 2018). Historical assessments of being unable to repay a debt have described it as being very difficult and painful for the debtor because their inability to restore themselves to parity (i.e. repay the debt) has been taken as an indication of there obviously being something wrong with them and that it must be their fault (Graeber, 2014, p. 137).

Contemporaneous contextualization of the indebted individual suggests that this vulnerable position is even worse and more widespread that previously assumed (Lazzarato, 2012; Ross, 2014). It is argued that in comparison with other predominant power relations, such as the capital-labor relationship, the creditor-debtor relationship is not as restricted to the space of the factory, but is much more transversal to society, that is, it is central to all social relationships (consumption, education, training, etc.; Charbonneau & Hansen, 2014). Further, today’s debt economy not only relies on an individual that is ‘morally invested in the promise to repay debt and the guilt this requires’ (Charbonneau & Hansen, 2014, p. 1040) in an unprecedented manner, but also on its injunction to become an ‘entrepreneur of the self’, imposing on the individual the costs and risk that the state and business externalize into society and are no longer willing to take upon themselves (Lazzarato, 2012, p. 93).

From a suicidological perspective, the vulnerability depicted in the sociohistorical assessments of the meanings of indebtedness, mirrors in several ways the core components of the suicidal process, when understood as being an escape from the self and the world (cf. Baumeister, 1990): (a) not being able to repay a debt is considered to be a form a failure, that is, a stressful life event that falls short of high standards and expectations; (b) the individual tends to blame themselves for failure to pay, meaning the failure produces internal attributions: ‘making one aware of oneself as inadequate, incompetent, guilty, or otherwise undesirable’ (Baumeister, 1990, p. 107); and (c) failure to pay is a painful experience filled with intensely negative emotions, including but not limited to, shame. Ultimately, it is a negative affect associated with this awareness of self-inadequacy that leads to the desire to escape, hence, the suicidal act. The fact that the debtor-creditor relationship can be seen as foundational to social life (Lazzarato, 2012) is also well suited to the notion of suicide as a form of escape, as escape from the self is always an escape from the social life that constitutes it (cf., Rojas, 2014), that is, the world (cf. Baumeister, 1990).

Empirically, and without downplaying the fact that the evidence base of the relationship between debt and suicide has been deemed to not be particularly strong (Haw et al., 2015), there is support for the notion that there is a suicidogenic effect in experiencing problems with debt repayment (Inoue et al., 2012; Lester & Yang, 2012). For example, it has been implied that merely having debts is not a sufficient criterion for being included as part of the group at risk of committing suicide (Arehart-Treichel, 2005), whereas research that addresses the suicidogenic effects at the other end of the debt continuum, for example, home evictions (due to payment problems) have, in turn, implied that the suicidogenic strain in question may have started before the actual execution of the enforced expulsion order (Rojas & Stenberg, 2016; Stack & Wasserman, 2007). Furthermore, recent accounts of the suicidogenic process behind suicide ideation and attempted suicide among debtors have localized the contact with a bailiff as being a key phase of the indebtedness process (Bond & Holkar, 2018).

### Debt and the Swedish Enforcement Authority

The Swedish Enforcement Authority is a government agency with a range of responsibilities with respect to debts, including, but not limited to, enforcement, debt collection, and injunctions to pay (Kronofogden, 2020; Regeringskansliet, 2021). Anybody with a legitimate payment claim can use it services, for example, the government, municipalities, companies, and private individuals. The Enforcement Authority can help a claimant obtain an official ruling that the debtor is obliged to pay a debt upon which a claimant can request the Enforcement Authority to attach earnings or other assets of the debtor. This is usually referred to as *execution* and it involves the debtor’s employer deducting money from the debtor’s salary, or the Enforcement Authority selling the debtor’s property (Kronofogden, 2020). It is not just salary that can be attached in this way: attachment of earnings can also be applied to other benefits and forms of remuneration such as pension, annuity, sickness benefit, parental benefit, unemployment benefit, and taxable reimbursement of costs (SOU, 2016:81).

The authority registers all debts that have been confirmed through a simplified payment procedure or a court order and in respect of which enforcement has been attempted but has been ineffective (Jørgensen, 2016).

## Method

### The study base – an exposed and a comparison group

The exposed group examined in this study includes all adults 18 to 64 years (at baseline), comprising women and men with a registration date for a debt in the Swedish Enforcement Authority (Kronofogden) register between 1 January 2015 and 31 December 2017.^1^ With the help of Statistics Sweden, the data from the exposed group in the Enforcement Authority register have been linked to several other national registers. This study makes use of the linkages made with the National Cause of Death Register, the National Patient Register and the Medicinal Drug Register, the Longitudinal Integration Database for Health Insurance and Labor Market Studies, the Population Statistics Register and the Geography Database, as well as the Register of Persons Convicted of Criminal Offences. These registers are administered by the National Board of Health and Welfare, Statistics Sweden, and the National Council for Crime Prevention, respectively.

For each of the individuals registered at the Enforcement Authority, a set of up to five controls were extracted (31 December, 2014) from the general Swedish population matched by age, gender, and region of residence. The data set for the comparison group contained the same information from the national registers as the data set for the group experiencing debt problems. The exposed group (i.e. the individuals in the group with a debt registered at the Enforcement Authority) were removed from the sample population before the comparison group was created.

The research project has been approved by the Swedish Ethical Review Authority (reference number: 2017/2227-31/5). All the data are stored at Statistics Sweden and have been made available to the author via its micro-data Online Access system.

### Analytical strategy

The available information on cause of death and the registration date at the Enforcement Authority in the study sample is from 2015 to 2017. This means that the follow-up period for those people who had a registration date for a debt during 2017 will be a maximum of 1 year. Consequently, the analysis of the exposed population is restricted to suicides that occurred within 1 year of the registration date at the Enforcement Authority, at any point from 2015 to 2017. In addition, comparison with the unexposed sample of the Swedish population is restricted to a 1-year follow-up period, more precisely, to death from suicide that occurred within 1 year of the comparison group being extracted (31 December, 2014). The control variables were measured at baseline for both the exposed and the comparison group, that is, during the calendar year preceding the start of the follow-up period.

Personal identification numbers identified as erroneous have been excluded, as cases of emigration during the study period. Moreover, the analysis only considers people for whom complete data are available on all the variables included in the models. However, for suicide cases that only contained information on the year and month of the date of death, the first day of the month was assigned as the day of death.

The relationship between the independent/control variables and suicide has been estimated using penalized maximum likelihood logistic regression (firthlogit). The loose-matching nature of the data under study allows it to be analyzed using unconditional logistic regressions (Kuo et al., 2018). The advantage of this form of analysis is that it is possible to obtain estimates for the matching variables (measured here on 31 December, 2014), by simply including them as regular control variables in the analysis. Further, firthlogit is a technique that is suited to dealing with situations in which the event of interest is rare (cf., Rojas, 2017; Rojas & Stenberg, 2016), which is the case here in the sense that the proportion of deaths from suicide in the data is lower than 5% (King & Zeng, 2001). Firthlogit is available as a sub-routine in STATA (Firth, 1993; Hilbe, 2009; StataCorp, 2019).

#### Dependent variable

Suicide is understood as being an external cause of death which, in accordance with the 10th revision of the International Classification of Diseases (ICD-10), has been defined in the Swedish Cause of Death Register as either intentional self-harm (X60–X84) or an event of undetermined intent (Y10–Y34; cf., Rojas & Stenberg, 2010).

#### Independent variable

Financial indebtedness is defined as having a registration date for a debt in the Swedish Enforcement Authority register at any point between 1 January 2015 and 31 December 2017.

#### Control variables

Although knowledge about the relationship between indebtedness and suicide is limited, we know more about the factors that immediately precede a registration for a debt at the Swedish Enforcement Authority (Kronofogden, 2008a; SOU, 2013:78; Vuleta, 2018). In order to be able to assess the extent to which being faced with registration at an enforcement authority for a debt can in itself be considered suicidogenic, we need to control for these factors.

According to the Swedish Enforcement Authority, the phenomenon of indebtedness is complex and includes several pathways, many of which have still not been empirically explored. Having said that, the Enforcement Authority has suggested that living on the margins could be a plausible common denominator among those people included in its registers. This is not to say that there are not cases in which criminal behavior, substance abuse, or mental illness, could be assumed to be a driving factor behind a debt problem. It is also known that men, young and middle-aged adults, big-city residents, single-person households, foreign-born persons, and individuals with a low level of education have tended to be overrepresented in the Enforcement Authority’s register (Kronofogden, 2008a, 2008b; SOU, 2013:78; Vuleta, 2018).

The control variables have been measured in line with the analytical strategy described above and are defined as follows. *Gender*: women/men; *Age*: year of birth. *Region of residence*: living in one of the three regions in Sweden that include the country’s three largest cities (Stockholm, Gothenburg and Malmö, respectively). *Place of birth*: Sweden/foreign born. *Single-person household*: single persons/all other family constellations (married families [including civil unions], cohabiting families, and single-parent families). *Education*: pre-upper secondary, upper secondary, and post-upper secondary education. *Criminal record*: convicted of a least one criminal offence over a 1-year period. Living on the margins is measured using two indicators: (i) *Social welfare recipiency* (received means-tested social assistance at least once over the course of 1 year) and (ii) *unemployment* (registered as unemployed by the relevant authorities for at least 1 day over the course of 1 year).

Because of the lack of specificity in terms of diagnoses of mental illness and debt problems in the literature, apart from substance abuse, the choice of control variables for this dimension has been based on the knowledge that mood disorders, substance abuse and schizophrenia have been found to be the three most common diagnoses in relation to completed suicide (Reutfors, 2010). *Mood disorder* is defined as being registered as being prescribed antidepressants (ATC-code: N06A) at least once over a 1-year period. *Substance abuse* is defined as being recorded in the Swedish inpatient care register with a mental or behavioral disorder due to psychoactive substance use as the main diagnosis (ICD 10: F10–F19) at least once over a 1-year period and *Schizophrenia* is defined as being recorded in the Swedish inpatient care register with schizophrenia or schizoaffective disorders as the main diagnosis (ICD 10: F20 and F25, respectively) at least once over a 1-year period.

## Results

The study base consists of an exposed group and a comparison group, comprising a total of 180,842 and 928,265 individuals, respectively (see Table 1). A total of 258 deaths by suicide are included in the analysis, of which 112 occurred in the exposed group and 146 in the comparison group. The proportion of people who committed suicide in the exposed group is approximately three times as large as the corresponding proportion in the comparison group. Apart from the matching variables of age, gender, and region of residence, the distributions of the control variables clearly differ between the exposed group and the comparison group, confirming the adverse conditions of those people who experienced financial indebtedness.

**Table 1.** Distribution of dependent and control variables included in the models by the group that had a registration date for a debt at the Swedish Enforcement Authority between 2015 and 2017 (exposed group) and the matched sample of the Swedish population (comparison group).

Variable	Respondents	
	Exposed group (*n* = 180,842)	Comparison group (*n* = 928,265)
Suicide (%)	0.06	0.02
Control variables		
Place of birth (%)		
Born in Sweden (reference category: foreign born)	71.26	82.21
Single-person household (%)		
Single (reference category: other family constellations)	22.33	17.20
Mood disorder (%)		
Prescribed antidepressants (reference category: other)	14.54	7.24
Substance abuse (%)		
Mental and behavioral disorders due to psychoactive substance use (reference category: other)	1.09	0.13
Schizophrenia (%)		
Schizophrenia or schizoaffective disorders (reference category: other)	0.15	0.05
Unemployment (%)		
Unemployed (reference category: other)	19.37	10.99
Social welfare recipiency (%)		
Received social assistance (reference category: other)	11.10	3.50
Education (%)		
Pre-upper secondary (reference category: post-upper secondary education)	22.76	12.91
Upper secondary (reference category: post-secondary education)	50.18	47.99
Criminality (%)		
Convicted of a criminal offence (reference category: other)	4.05	0.74
Age (mean)		
Year of birth	1977.88	1977.01
Gender (%)		
Men (reference category: women)	60.75	61.07
Region of residence (%)		
Living in a big city (reference category: other)	56.33	56.75

The results from the penalized maximum likelihood logistic regression analysis are presented in Table 2. In Model 1, we see that indebtedness is significantly related to suicide, with a corrected OR of 3.94. In other words, people who had a registration date for a debt at the Swedish Enforcement Authority at any point between 1 January 2015 and 31 December 2017 were approximately four times more likely to commit suicide than people who had not been exposed to this experience. As can be seen in Model 2, this relationship remained practically unchanged when adjusted for age, gender, place of birth, region of residence, and single-person household. When mood disorders, substance abuse, and schizophrenia were included in the analysis, the effect of indebtedness remained significant but decreased in strength, from an OR of 3.84 to 2.77 (see Model 3). In the final model, four additional control variables were introduced into the analysis (unemployment, social welfare recipiency, education, and criminality). The effect of indebtedness remained significant but decreased slightly in strength, OR = 2.50.

**Table 2.** Penalized maximum likelihood logistic regression of financial indebtedness and suicide in Sweden 2015 to 2017.

Variable	Model 1 crude OR [95% CI]	Model 2 adjusted OR [95% CI]	Model 3 adjusted OR [95% CI]	Model 4 adjusted OR [95% CI]
Independent variable				
Financial indebtedness				
Debt registered at the Enforcement Authority (reference category: other)	3.94 [3.08–5.04]*	3.84 [2.99–4.92]*	2.77 [2.14–3.60]*	2.50 [1.92–3.27]*
Control variables				
Age				
Year of birth (continuous)		0.98 [0.97–0.99]*	0.98 [0.98–0.98]*	0.98 [0.97–0.98]*
Gender				
Men (reference category: women)		2.03 [1.52–2.72]*	2.45 [1.82–3.30]*	2.32 [1.72–3.13]*
Place of birth				
Born in Sweden (reference category: foreign born)		1.52 [1.07–2.16]*	1.31 [0.93–1.86]	1.42 [0.99–2.03]
Region of residence				
Living in a big city (reference category: other)		0.87 [0.68–1.11]	0.86 [0.67–1.10]	0.90 [0.70–1.15]
Single-person household				
Single (reference category: other family constellations)		3.12 [2.44–4.00]*	2.54 [1.98–3.27]*	2.51 [1.95–3.23]*
Mood disorder				
Prescribed antidepressants (reference category: other)			4.59 [3.50–6.03]*	4.43 [3.37–5.81]*
Substance abuse				
Mental and behavioral disorders due to psychoactive substance use (reference category: other)			7.59 [4.78–12.03]*	5.69 [3.50–9.24]*
Schizophrenia				
Schizophrenia or schizoaffective disorders (reference category: other)			8.06 [3.55–18.22]*	7.78 [3.42–17.68]*
Unemployment				
Unemployed (reference category: other)				1.15 [0.81–1.63]
Social welfare recipiency				
Received social assistance (reference category: other)				1.44 [0.94–2.22]
Education				
Pre-upper secondary (reference category: post-upper secondary education)				1.27 [0.87–1.85]
Upper secondary (reference category: post-secondary education)				1.31 [0.97–1.77]
Criminality				
Convicted of a criminal offence (reference category: other)				2.49 [1.54–4.05]*
Suicides	258	258	258	258
Total study population (*n*)	*n* = 1,109,107	*n* = 1,109,107	*n* = 1,109,107	*n* = 1,109,107

* Statistically significant (at the 5% level).

In Table 3, the models in Table 2 have been replicated, dividing the exposed group into people who were also subject to an attachment of earnings, property foreclosure, and/or eviction (*n* = 23,965; suicides = 24) during the follow-up period following the registration date for a debt at the Enforcement Authority (and before the date of death by suicide) and people who were not (*n* = 156,877; suicides = 88). The objective here was to examine the level of sensitivity of the final results to common forms of execution actions taken by an enforcement agency. The effect of being registered at the Enforcement Authority for a debt remained practically the same in terms of both size and statistical significance (Compare Model 4 in Table 2 with the sensitivity test of Model 4 in Table 3), irrespective of the different types of execution actions taken by the agency.

**Table 3.** Penalized maximum likelihood logistic regression of financial indebtedness and suicide in Sweden 2015 to 2017, distinguishing between common types of execution actions taken by the Enforcement Authority.

Variable	Sensitivity test for Model 1 crude OR [95% CI]	Sensitivity test for Model 2 adjusted OR [95% CI]	Sensitivity test for Model 3 adjusted OR [95% CI]	Sensitivity test for Model 4 adjusted OR [95% CI]
Independent variable				
Financial indebtedness				
Debt registered at the Enforcement Authority without attachment of earnings/foreclosure of property/eviction	3.58 [2.75–4.66]*	3.58 [2.74–4.67]*	2.71 [2.06–3.58]*	2.43 [1.83–3.22]*
Debt registered at the Enforcement Authority with attachment of earnings/foreclosure of property/eviction (reference category: other)	6.48 [4.23–9.95]*	5.27 [3.40–8.16]*	3.12 [1.99–4.90]*	2.89 [1.84–4.53]*
Control variables				
Age				
Year of birth (continuous)		0.98 [0.97–0.99]*	0.98 [0.96–0.99]*	0.98 [0.97–0.98]*
Gender				
Men (reference category: women)		2.05 [1.53–2.74]*	2.46 [1.83–3.31]*	2.33 [1.73–3.14]*
Place of birth				
Born in Sweden (reference category: foreign born)		1.52 [1.07–2.15]*	1.32 [0.93–1.86]	1.42 [0.99–2.03]
Region of residence				
Living in a big city (reference category: other)		0.88 [0.69–1.12]	0.86 [0.67–1.10]	0.90 [0.71–1.16]
Single-person household				
Single (reference category: other family constellations)		3.08 [2.40–3.94]*	2.53 [1.96–3.26]*	2.49 [1.93–3.21]*
Mood disorder				
Prescribed antidepressants (reference category: other)			4.58 [3.47–6.03]*	4.41 [3.36–5.79]*
Substance abuse				
Mental and behavioral disorders due to psychoactive substance use (reference category: other)			7.52 [4.74–11.96]*	5.64 [3.47–9.16]*
Schizophrenia				
Schizophrenia or schizoaffective disorders (reference category: other)			7.97 [3.52–18.06]*	7.68 [3.38–17.48]*
Unemployment				
Unemployed (reference category: other)				1.15 [0.82–1.63]
Social welfare recipiency				
Received social assistance (reference category: other)				1.45 [0.95–2.22]
Education				
Pre-upper secondary (reference category: post-upper-secondary education)				1.26 [0.86–1.84]
Upper secondary (reference category: post-secondary education)				1.30 [0.97–1.76]
Criminality				
Convicted of a criminal offence (reference category: other)				2.51 [1.54–4.08]*
Suicides	258	258	258	258
Total study population (*n*)	*n* = 1,109,107	*n* = 1,109,107	*n* = 1,109,107	*n* = 1,109,107

* Statistically significant (at the 5% level).

## Discussion

Reducing the risk of suicide is an imperative public health issue (WHO, 2014). In fact, the National Action programme for Suicide Prevention, includes a zero vision for suicide, stating that ‘no one should have to end up in a situation of such vulnerability that suicide is seen as the only way out’ (Folkhälsomyndigheten, 2016, p. 3). Nevertheless, approximately 1,500 people in Sweden still die by suicide each year (Folkhälsomyndigheten, 2019). Apart from monitoring its prevalence and trends, a key element of the work toward reducing suicide, as well as developing, implementing, and evaluating effective strategies and interventions, is identifying its related risk and protective factors, that is, conducting research in order to establish why suicide occurs and whom it affects (WHO, 2014). The current study has set out to deepen our understanding of the extent to which financial indebtedness constitutes one of these vulnerable positions that may enhance an individual’s risk of committing suicide.

Debt has tended to be overlooked in the suicidological literature (Richardson et al., 2013). This is true to the extent that it is being referred to as a silent killer (Bond & Holkar, 2018). Furthermore, the evidence base showing a detrimental relationship between unmanageable debt and suicide appears to be overrepresented by studies based on Asian countries (particularly China and Japan; Haw et al., 2015; Richardson et al., 2013). This has even prompted some scholars to question the extent to which this devastating reaction to debt might be culture specific (Lester & Yang, 2012).

The results of this study challenge this view, at least as far as Sweden is concerned. Debt repayment problems, as registered at the Enforcement Authority, were found to be related to suicide, adjusted for multiple background variables. Furthermore, the nationwide large-scale register data employed in this study also made it possible to establish that the suicidogenic effects of indebtedness is not reducible to a question of execution actions taken by the authority (i.e. attachment of earnings/foreclosure of property/eviction), suggesting that being unable to repay a debt would be a sufficiently vulnerable position in terms of suicidality. This is also in line with the theoretical assumptions outlined at the outset of this study, which highlight that in a context in which the debtor-creditor relationship is transversal to society and constituent of social life – requiring individuals to be morally invested in the promise to repay debt and the guilt that this implies (cf., Lazzarato, 2012) – it is not difficult to envisage that breaking with the obligation inherent to this relationship, that is, not being able pay a debt, may be a very painful experience filled with intensely negative emotions and self-blame, leading to a desire to escape, and ultimately, the suicidal act (cf., Baumeister, 1990).

Having said that, the possibility that debt problems probably act as a suicidogenic risk factor in more than one modern Western country, without receiving much attention, could very well relate to historical and cultural aspects. In fact, the relational perspective on suicide, which is alluded to by the literature on the subject (Chan et al., 2005; Lester & Yang, 2012), that is, the idea that suicide is an act in relation/response to social dynamics (such as the debtor-creditor relationship) that have become unacceptable, has been relatively neglected in the modern Western world (Lee & Kleinman, 2003; Wu, 2010). Furthermore, the overall silence surrounding the link between debt problems and suicidal behavior, which independent charitable organizations are currently trying to highlight (Bond & Holkar, 2018), is in itself indicative of a normative disinterest, within these societies, to view debt commitment difficulties as a societal problem. This might perhaps be related to the strong individual stigma that is attached to the inability to repay a debt (Di Leo et al., 2018; Walker, 2012), in Europe, at least, the tendency has been to focus on moral issues rather than on the shortcomings in the market with regard to understand indebtedness (SOU, 2013:78). After all, taboo, stigma, shame, and guilt play a key role in obscuring different types of behavior (WHO, 2014).

### Limitations

Two main methodological considerations should be borne in mind when interpreting the results of this study. First, because the study is entirely based on register data, there is a lack of self-reported information on confounders that may have influenced the results (Thygesen & Ersbøll, 2014), for example, attitudes toward debt. However, capturing representative and sizeable groups of individuals with this type of debt problem through traditional surveys and following them up in terms of death by suicide has thus far proven to be very difficult, which makes register-based studies of the kind presented here very important (Thygesen & Ersbøll, 2014).

Second, as may be the case in situations in which rare events are endemic to the data (Rojas & Stenberg, 2010, 2016) the study has been unable to control for possible multiplicative effects that might be present between debt problems and one or more of the background variables included in the final model. However, the research question has only been concerned about the main effects. Furthermore, as long as the evidence base for including debt problems as an important social risk indicator of suicide in its own right remains under scrutiny, the strategy adopted in this study will continue to be important. After all, before we can address the complexities of a given social problem, we first need to acknowledge that the problem exists.

## Conclusion

Financial indebtedness has a detrimental impact on an individual’s risk of committing suicide. This effect is statistically independent of other common suicidogenic risk factors present prior to the date of registration of a debt at the Swedish Enforcement Authority, including, but not limited to, socioeconomic (e.g. unemployment), mental health (e.g. depression), and behavioral (e.g. criminality) risk factors. Furthermore, the detrimental effect of non-payment of debt is not reducible to common types of execution actions taken by an enforcement agency (e.g. attachment of earnings). Although a few other studies have recognized this connection, this is the first study to examine the relationship between debt problems and suicide, using large-scale register data from multiple agencies for an entire country. Thus, the experience of being registered for debt at an enforcement authority should be regarded as a stressful life event in its own right. Having said that, further studies are needed in order to confirm these findings.

## Notes

1. The information from the Swedish Enforcement Authority database was extracted January 11, 2018. An individual who has been registered at the Swedish Enforcement authority may be kept in the register for five years but can be removed earlier. In practice a debtor is kept in the register a minimum of three years after that his or her latest matter has been closed by the Authority (SOU, 2013:78).
